# Current and Emerging Treatment Options in Sinus and Nasal Diseases: Surgical Challenges and Therapeutic Perspectives

**DOI:** 10.3390/jcm12041485

**Published:** 2023-02-13

**Authors:** Xiao-Ying Zhao, Ming Chen, Lei Cheng

**Affiliations:** 1 Department of Otorhinolaryngology-Head and Neck Surgery, The Second Affiliated Hospital, School of Medicine, Zhejiang University, Hangzhou 310009, China; 2 Department of Otorhinolaryngology & Clinical Allergy Center, The First Affiliated Hospital, Nanjing Medical University, Nanjing 210029, China

Chronic rhinosinusitis (CRS), one of the most prevalent health problems worldwide, is defined as a chronic inflammation of the nasal and paranasal sinuses mucosa persisting for more than 12 weeks [1]. It has been estimated that CRS affects up to 12% of the population in America [2], 10% in Europe, and 8% in China [3], significantly impacting patients’ quality of life [4]. Clinically, patients with CRS are characterized by main symptoms, including nasal blockage, purulent rhinorrhea, hyposmia or anosmia, and facial pain or pressure, while a minor number of patients suffer from headache, fatigue, dizziness, sleep disturbance, and ear pain or pressure as well [1].

CRS is traditionally classified into two major categories—CRS with nasal polyps (CRSwNP) or without nasal polyps (CRSsNP) based on phenotype [5]. However, the classification criteria for CRS is further updated according to the European Position Paper on Rhinosinusitis and Nasal Polyps 2020 (EPOS 2020), anatomically dividing both primary and secondary CRS into localized and diffuse disease [1]. Regarding the pathophysiology, emerging evidence has demonstrated that differential underlying immune response patterns may explain the heterogeneity of CRS [6], suggesting clinicians should emphasize more on CRS endotypes such as type 2 inflammation and non-type 2 inflammation [7]. The former is induced by Th2/Tc involving various cytokines such as IL-4, IL-5, IL13, and a predominant presence of eosinophil infiltration [8]. Immunoglobulin E and eosinophilia in nasal mucosa are demonstrated as the features of type 2 inflammation [9]. In comparison, the latter is mediated by Th1/Th17, with increased levels of IFN-γ, IL-17, IL-22, and IL-23 [10].

Treatment options for CRS recommended by guidelines generally include medical management and surgical intervention, aiming to control the inflammation and reconstruct the physiological function of the nasal/sinus cavity [1,11]. Plenty of CRS patients still behave refractory to maximal medical therapy (including antibiotics, corticosteroids, antihistamines, decongestants, and saline irrigation), and surgical treatment such as functional endoscopic sinus surgery (FESS), steroid-eluting sinus stent (SES) and techniques of nasalization should be taken into consideration afterward [12,13,14].

FESS aims to remove nasal polyps, creates a sinus cavity, drain the purulent discharge and facilitates mucociliary clearance [1]. Significant symptom improvements have been reported among the CRS patients who were treated with FESS. However, a minority of patients with CRSwNP who are characterized by type 2 inflammation still suffer from recurrence after surgery [14]. A European cohort study reports that at least 40% of CRS patients remain uncontrolled for 3–5 years post-surgery, and 17% of enrolled CRS subjects have a revision surgery [15]. Patients who remain resistant to adequate sinus surgery and corticosteroid/antibiotics for two courses are considered to be refractory chronic rhinosinusitis (RCRS) [16]. As for RCRS and CRSwNP dominated by type 2 inflammation, new surgical techniques have been developed to improve the postoperative outcomes and reduce the disease recurrence.

Clinical guidelines suggest persisting drug therapy for at least one month before shifting to surgical treatment [17]. FESS is a frequently utilized treatment in the management of CRS, which applies to most CRSwNP patients. It is characterized by clearer vision, less blood loss, earlier recovery, less trauma, and fewer complications. The principle of FESS in treating CRS is to enlarge sinus ostia, restore adequate aeration of sinuses, and improve mucociliary transport, providing better access for medical therapies to the sinus mucosa [18]. Despite the straightforward notion behind FESS, the anatomical variability and the heterogeneity of diseases remain challenging for the surgeon in every case.

The extent of the surgery depends on the degree of the seriousness of the disease [19]. It is well agreed that FESS provides an effective treatment for CRS dominated by the type 1 inflammation and non-RCRS with less damage and fewer difficulties [20], while more aggressive FESS, known as the techniques of nasalization and reboot, may offer a lower level of recurrence in patients with type 2 inflammation or RCRS. Nasalization is referred as a radical ethmoidectomy, while Reboot remove all sinonasal polyps and mucosa down to the periosteum, so Reboot requires more complete debridement of inflamed sinus mucosa than nasalization [21,22], surgery combined with steroid drug stent shows significant improvement in reducing the risk of recurrence [23].

Nasalization is a radical ethmoidectomy referred to as the maximally opening of the ethmoid, maxillary, sphenoid, and frontal sinuses, and completely/partially remove the middle turbinates, aiming to resect non-olfactory mucosa as completely and safely as possible [22]. Sinus respiratory function and the physiological role of the ostia should be conserved based on the developed conception of nasal pathology, in which CRS is defined as an inflammation involved non-olfactory mucosa specifically. Nasalization opens all sinuses largely to reshape a massive nasal cavity which ensures the assessment of topical steroids [22,24]. The significantly better functional outcomes for nasalization than for functional ethmoidectomy were demonstrated by the first comparative study in 1997 [24]. In a 5-year follow-up study, comparing functional results after nasalization and ethmoidectomy, the recurrence rates of nasalization were significantly lower than that of functional ethmoidectomy (22.7% vs. 58.3%) [25].

To achieve a nasalization procedure, otorhinolaryngologists are recommended to (i) enlarge sinus ostium and sphenoidotomy, (ii) perform the middle turbinectomy, (iii) safely resect as much ethmoid mucosa as possible, following the ethmoid wall, and (iv) progress towards the frontal sinus centripetally during the dissection [26]. Patients suitable for nasalization are more likely to be involved with asthma, hyposmia, and recurrent CRS, which indicates nasalization seems well-adapted to the treatment of RCRS and type 2-dominated CRS. Surgery, as well as systemic steroids, probably act by depleting the ethmoid reservoir of eosinophils [26,27].

There are two different reboot techniques: a partial reboot surgery involves the resection of all sinonasal polyps and mucosa down to the periosteum, and a complete reboot surgery requires an extra Draf III procedure [21]. It aims to remove all inflamed sinus mucosa and allow the re-epithelization of functional nasal mucosa for CRSwNP dominated by type 2 inflammation [28]. A retrospective study by Alsharif et al. [29] discovered a lower recurrent rate and longer recurrent time for the reboot surgery group compared to the FESS group, indicating a promising future to benefit more CRS patients. However, while lacking evidence of successful re-epithelization after extensive mucosa ablation, nor that the inferior turbinate or nasal septum mucosa can extend to the surgical cavity, the concept of reboot remains controversial. In addition, appropriate instruments fail to reach the top of the frontal sinus and the deep part of the maxillary sinus; therefore, complete removal of sinus mucosa can only be performed at a theoretical level. It is important to note that the massive trauma of sinus mucosa may lead to a demuconization reaction, making it more challenging than recurrent polyps. Given the limited and inconsistent studies, the evidence of the benefit of reboot use is still uncertain, and therefore recommendations are lacking.

On a practical note, specific intraoperative procedures help to make surgery more effective and safer. Concerned about postoperative adhesion, edema, and polyp reformation, persistent medical treatment is recommended to reduce the inflammation load after FESS. SES is a promising solution designed to be implanted in the sinuses and continuously release anti-inflammatory medicine to prevent the above concerns [30]. Previous studies have revealed that the effects of anti-inflammation agents of SES can be maintained for 3 months, contributing to a significant improvement in post-operative outcomes and endoscopic evaluation compared with placebo or traditional topical/oral steroids [31]. According to a meta-analysis, seven randomized controlled trials of 444 participants confirmed the potential benefits of SES for reduced post-operative intervention, inflammation, recurrent polyposis, and frontal sinus ostia restenosis [32]. Despite the positive effect of intraoperative steroid delivery techniques for CRS, extra costs and concerns over side effects of steroid make it less favorable [33]. Further cohort studies are warranted to resolve the following concerns. Confirming the sufficiency and efficiency of the drugs delivered by SES will be of value to optimize surgical results. The investigation into whether the extravasated blood attached to SES will affect efficiency is also an interesting topic. Furthermore, the influence of SES displacement caused by nasal packing after FESS is expected to be investigated.

Balloon catheter sinuplasty (BCS) functions effectively by sending a deflated balloon into the sinuses with the guidewire, then enlarging the ostia physically to flush and drain the sinuses by balloon inflation [34]. It is worth noting that BCS tends to restore the sinus ventilation at the least cost of tissue trauma since no bone or tissue is removed during the procedure. Therefore, BCS of the maxillary and frontal sinuses has been considered as a minimally invasive and safe procedure, which is reasonable for pediatric CRS specifically as a safer and more effective treatment compared to FESS. In a study on the efficacy of functional endoscopic dilatation sinus surgery (FEDS) compared with FESS in managing CRS, Acher et al. [34] reported similar positive results in two groups. Interestingly, Chaaban et al. [35] found that the elderly population over 60 is more likely to take in-office BCS than younger patients. Ideally, BCS seems to be potentially beneficial for CRS patients as an alternative tool. Simple BCS is adapted explicitly to CRSsNP, while the combination with FESS is favorable for its incremental safety and effectiveness [35].

CRS is a common disease among the pediatric population and causes a reduction in quality of life. The surgical option should be considered when maximal medical therapies fail in 3–6 weeks. Compared to adult patients with CRS, refractory pediatric CRS have different surgical options, including adenoidectomy, pediatric endoscopic sinus surgery (PESS, mainly involved with maxillary antrostomy and anterior ethmoidectomy), and BCS. Adenoidectomy is the first choice for non-asthmatic children aged ≤ 6 years with pediatric refractory CRS [36]. For older children with particular conditions (asthmatic children, or children with challenging systemic/local disease) who are refractory to medicine and adenoidectomy, traditional FESS should be considered as a potentially effective treatment [37]. The incidence of nasal polyps in children is estimated to be around 0.1%, split into five subgroups [38]. Large choanal polyps and antrochoanal polyps (also known as Killian polyps) referred to as isolated polyps, are frequently seen in children. FESS seems favorable for these two groups with positive results. Another two phenotypes are more likely to be multiple in polyps and rare in the pediatric population, including CRSwNPs driven by non-eosinophil-dominated or eosinophil-dominated inflammation. They appear to have a higher rate of recurrence after FESS, contrary to Killian polyps, and it remains controversial whether techniques of nasalization should be applied for pediatric RCRS. Finally, the last phenotype group refers to nasal polyps that develop in terms of systemic diseases such as cystic fibrosis and primary ciliary dyskinesia.

Advances in surgical treatment, ranging from the least extensive polyp extraction to the most extensive nasalization and reboot techniques, have significantly improved the management of CRS. As the surgical trauma involved grows, however, the safety and outcomes of reboot surgery remain to be evaluated. Steroid stent seems well adapted to all kinds of procedures, which is optional for every FESS patient considering its high cost. In conclusion, the management of CRSwNP needs to be individualized, flexible, and comprehensive, and a regular follow-up should be settled.

## Data Availability

Not applicable.
